# Editorial: In celebration of women in science: glycoscience

**DOI:** 10.3389/fmolb.2023.1215738

**Published:** 2023-05-15

**Authors:** Maren Roman, Preethi L. Chandran, M. Florencia Haurat

**Affiliations:** ^1^ Virginia Tech, Blacksburg, VA, United States; ^2^ Department of Biochemistry and Molecular Biology, College of Medicine, Howard University, Washington, DC, United States; ^3^ Department of Chemical Engineering, College of Engineering and Architecture, Howard University, Washington, DC, United States; ^4^ United States Food and Drug Administration, Silver Spring, MD, United States

**Keywords:** hyaluronan, glycolipids, cellulose nanocrystal films, glycosylation, sialyltransferase

The stories of historic female scientists are inspiring. They show a deeply rooted passion for advancing our understanding of the natural world but also tenacity and perseverance in a male-dominated environment. The determination of these women to pursue their calling in the face of repudiation and hindering gender norms paved the way for modern-day female researchers.

While barriers for women to enter research professions are lower now than they were in the past, retention and advancement of female researchers are still comparatively low. Accordingly, female leadership in science is a relatively recent phenomenon. Inclusive and supportive research ecosystems have thrived from the representation of women in advanced and leadership positions.

We are pleased to present the inaugural Frontiers in Molecular Biosciences “Women in Glycoscience” Research Topic of articles. The series seeks to spotlight the work of female thought leaders who are shaping the field of glycoscience as well as that of female researchers in early stages of their careers. We proudly present this article Research Topic to inspire the next-generation of female scientists from diverse and deterrent backgrounds.

The science of glycosylation *per se* is pervasive, leaving strong footprints on developments in diverse fields, such as disease mechanisms, biotherapeutics, material science, biomechanics, and sustainability. Working with sugars poses unique challenges for conventional probing, synthesis, and structure analysis techniques, yet leading female scientists continue to overcome persistent challenges with innovative approaches. The work of recent Nobel laureate Carolyn Bertozzi on imaging of cellular glycosylation, which ushered in a new field of bio-orthogonal click chemistry (Bertozzi, 2023), is a prime example of the impact of female scientists on the field.

This inaugural “Women in Glycoscience” article Research Topic contains a sampling of the diverse contributions of women to the field.

Novel assay techniques such as those presented by Sin et al. continue to enhance perspective on glycan-glycan and protein-glycan interactions, and identifying molecules that confound these interactions.

The work by Manthrirathna et al. highlights the persistent problems associated with integrating the ligand-binding and material science delivery challenges in using sugar-functionalized biomolecules as therapeutics and vaccines (glycobiology).

Nilsson et al. conducted a design of experiment (DoE) study of cellulose nanocrystal (CNC) films containing multiple plasticizers and developed empirical models to predict the mechanical and optical characteristics of the films. The developed modeling framework paves the way for commercial CNC film applications by enabling the prediction of film compositions that yield desired film properties.

Bacteria can synthesize a variety of glycan structures that cannot be found in eukaryotes. Szymanski reviews the field of microbial glycobiology using the foodborne pathogen *Campylobacter jejuni* as the model organism.

Aberrant protein glycosylation has been linked to various disease states. For example, the upregulation of the Golgi-sialyltransferase ST6Gal1 (β-Galactoside α-2,6-Sialyltransferase 1) has been observed in many malignancies, and its activity impacts cancer hallmarks. GC et al. summarize the oncogenic signaling pathways and targets affected by ST6Gal1.
